# Biomarkers and Alzheimer’s disease: a bibliometric analysis

**DOI:** 10.3389/fnagi.2024.1456824

**Published:** 2024-10-30

**Authors:** Linyi Yang, Jingyan Zeng, Linlin Li, Yunwei Zhang

**Affiliations:** ^1^Department of Neurology, Suining Central Hospital, Suining, China; ^2^Department of Respiratory and Critical Care Medicine, Suining First People’s Hospital, Suining, China

**Keywords:** biomarkers, Alzheimer’s disease, visual analysis, bibliometrics, research status, growing trend

## Abstract

**Objective:**

The diagnosis and treatment of biomarkers in Alzheimer’s disease has emerged as a prominent topic within Alzheimer’s disease research. In this paper, we conducted a bibliometric analysis of data from a wide range of literature in this field to enhance the in-depth understanding of this area.

**Method:**

The core collection of the Science Citation Index database (web of science) was used to search for relevant literature in the above fields from 1 January 2006 to 14 November 2022 and Citespace software was used to visualize and analyze the literature data.

**Results:**

A total of 1,138 papers were included, of which the United States ranked first with 607 papers and China ranked 6th in the world with 84 papers. The value of mediational centrality is 0.49 in the United States and 0.05 in China. In terms of the number of articles published by the research authors, the Swedish scholar Blennow Kaj ranks first with 82 articles published, and the scholars who rank second and third are Zetterberg Henrik (78 articles) and Morris John C (64 articles), respectively; in terms of the mediational centrality, the American scholar Trojanowski John Q ranked first in the world with 0.1, and the second and third ranked scholars were Blennow Kaj (0.09) and Zetterberg Henrik (0.06) respectively. Scholar JACK CR ranked first with 377 citation frequency. The journal NEUROLOGY is ranked first with 943 citations.

**Conclusion:**

In recent years, global research in the field of biomarkers related to Alzheimer’s disease has shown signs of softening, and the momentum of research has slightly diminished. However, this trend does not imply a decline in the quality of research. It is essential to enhance collaboration among countries, major research institutions, and scholars, with a particular emphasis on fostering international partnerships in the future.

## 1 Introduction

Alzheimer’s disease (AD) is clinically characterized by progressive cognitive and behavioral impairments. Early diagnosis of Alzheimer’s disease is a primary focus of clinical research, and the identification of biomarkers such as Aβ1-40, Aβ1-42, T-tau, and P-tau has established a robust diagnostic foundation for clinicians. Bibliometrics, a discipline that employs mathematical and statistical methods to quantitatively analyze literature, reveals patterns related to the quantity, distribution, influence, and developmental trends of scholarly work. This field aids in understanding the current research landscape, emerging trends, significant issues, and key contributors within a specific domain, ultimately providing valuable insights for scientific research decision-making. As the global population continues to grow, the incidence of Alzheimer’s disease is anticipated to rise, placing an even greater burden on healthcare systems. Notably, bibliometric analyses focused on biomarkers for the diagnosis and treatment of Alzheimer’s disease have not been documented in existing literature databases. In this paper, we conducted a literature review using the Web of Science Core Collection for visual analysis and interpretation, aiming to understand the current status and developmental trends in this research area, with the goal of identifying additional potential biomarkers and valuable research directions.

## 2 Materials and methods

### 2.1 Data collection

A data search of the web of science core collection was conducted on 2022-11-14. The search utilized the terms “biomarker” and “Alzheimer(s) disease” as subject terms. The search formula employed was TS = (Alzheimer OR Alzheimer’ disease) AND TS = (biomarker) AND LA = (English) AND DT = (Article OR Review) AND PY = (2006–2022).

### 2.2 Data extraction and processing methods

Citespace is the most mature and frequently used software for bibliometric analysis. In this paper, Citespace and Tableau public software were used for visual mapping analysis. The retrieved literature is exported, the content is selected as “full record of cited literature,” the format is selected as “full text,” and Notepad++ software is used to process the original data, supplement the missing information and integrate the same information.

### 2.3 Data analysis

The data were mapped using GraphPad Prism9, with a time span of 2006–2022, a time slice of 1 year, and a threshold of “first 50 nodes per slice” in the Citespace software.

## 3 Results

### 3.1 Characteristics of the included literature

**Figure 1:**
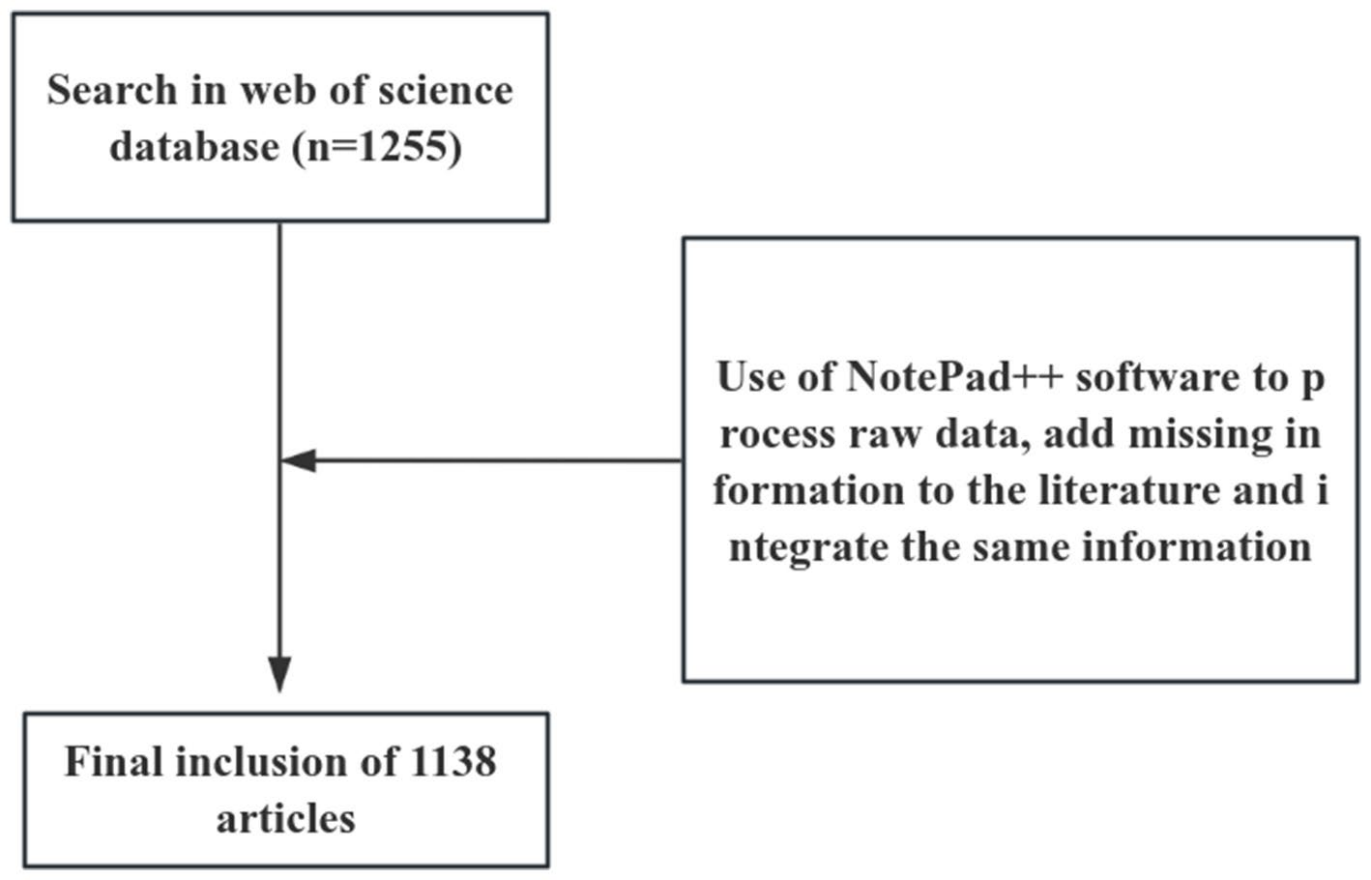
Flowchart of relevant research literature search in web of science database.

A total of 1,255 relevant documents were retrieved, and 1,138 documents were finally screened (Figure 1), with an overall h-index of 126, an average citation frequency of 54.34 per article, a total of 61,945 citations, and a total of 59,924 citations after removing self-citations.

### 3.2 Analysis of biomarker postings in Alzheimer’s disease research

**Figure 2:**
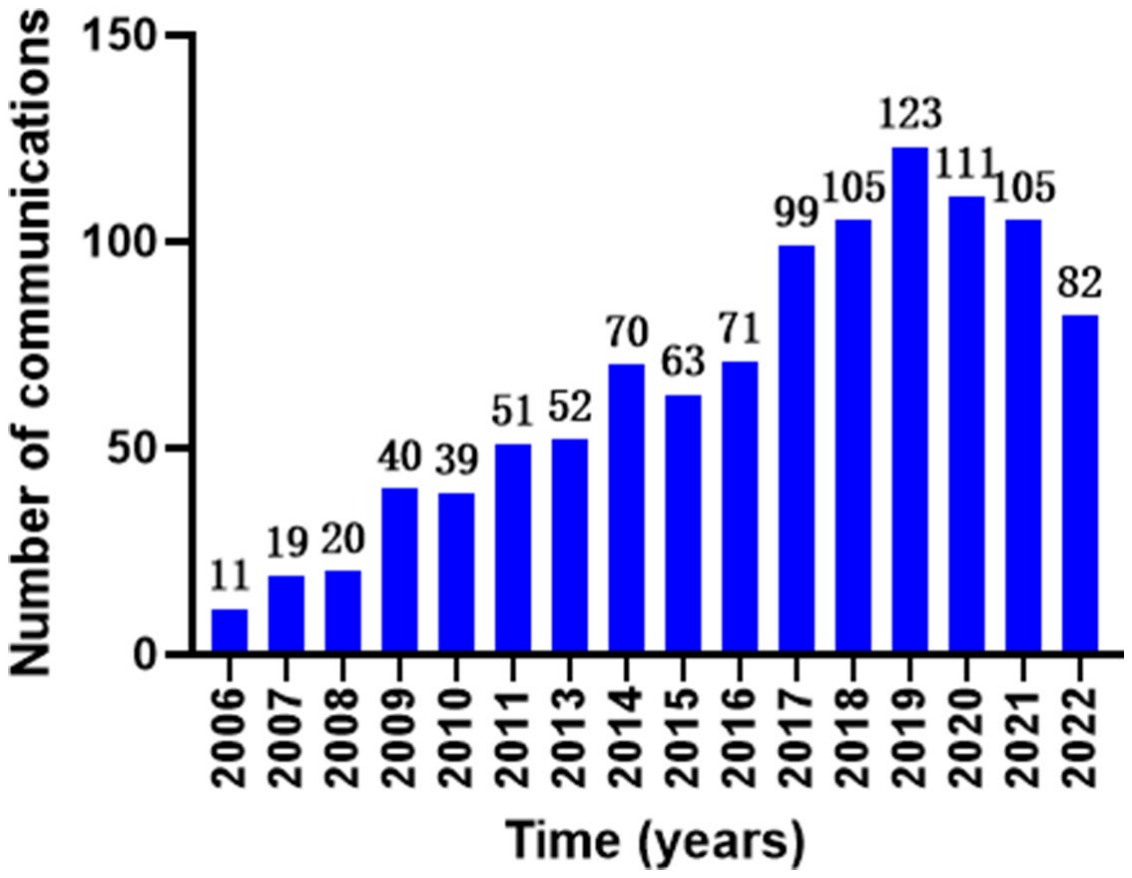
Annual number of publications on biomarkers and Alzheimer’s disease in the web of science database.

The current status of the relevant research areas can be reflected to some extent by mapping the annual volume of publications (Figure 2).

### 3.3 Country distribution of biomarker and Alzheimer’s disease related studies

**Figure 3:**
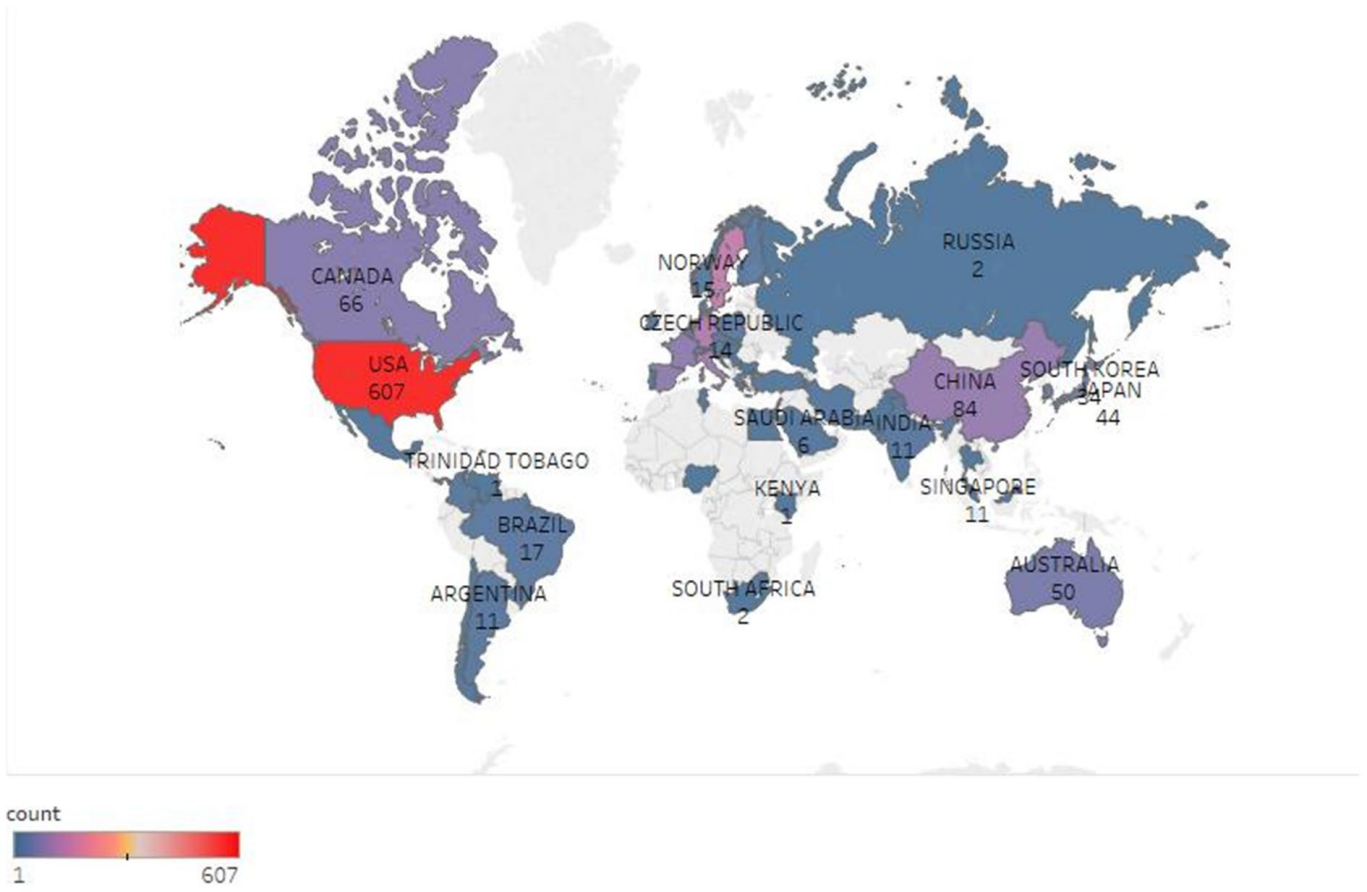
Geographic distribution of biomarkers and Alzheimer’s disease in the web of science database.

**Figure 4:**
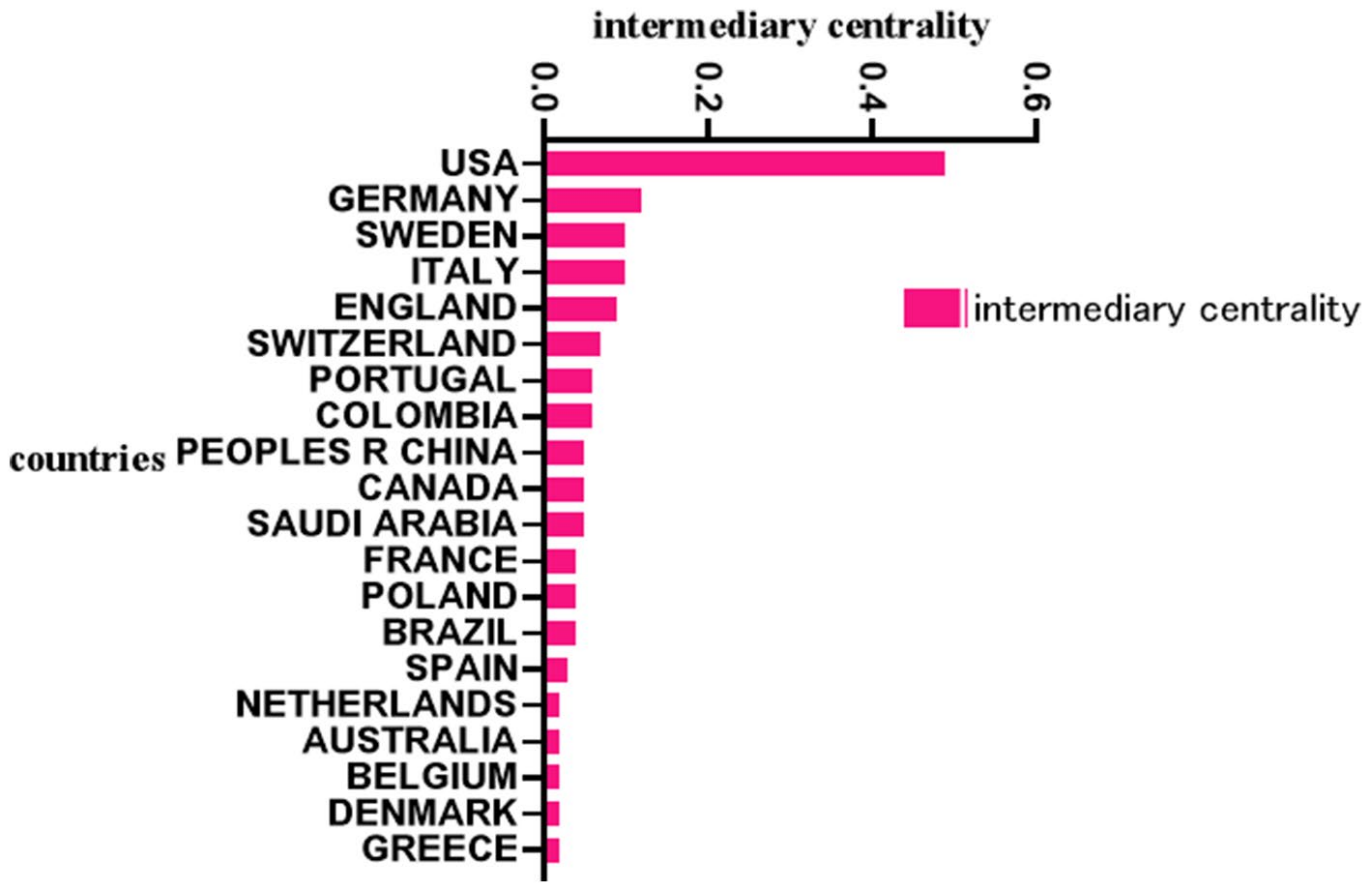
National mediated centrality of biomarker and Alzheimer’s disease related studies in web of science database (TOP20). Intermediary centrality is a reference used by the Citespace software to measure the importance of a metric, with centrality over 0.01 generally defined as a key metric. Higher values of intermediary centrality suggest greater influence of the corresponding country.

**Figure 5:**
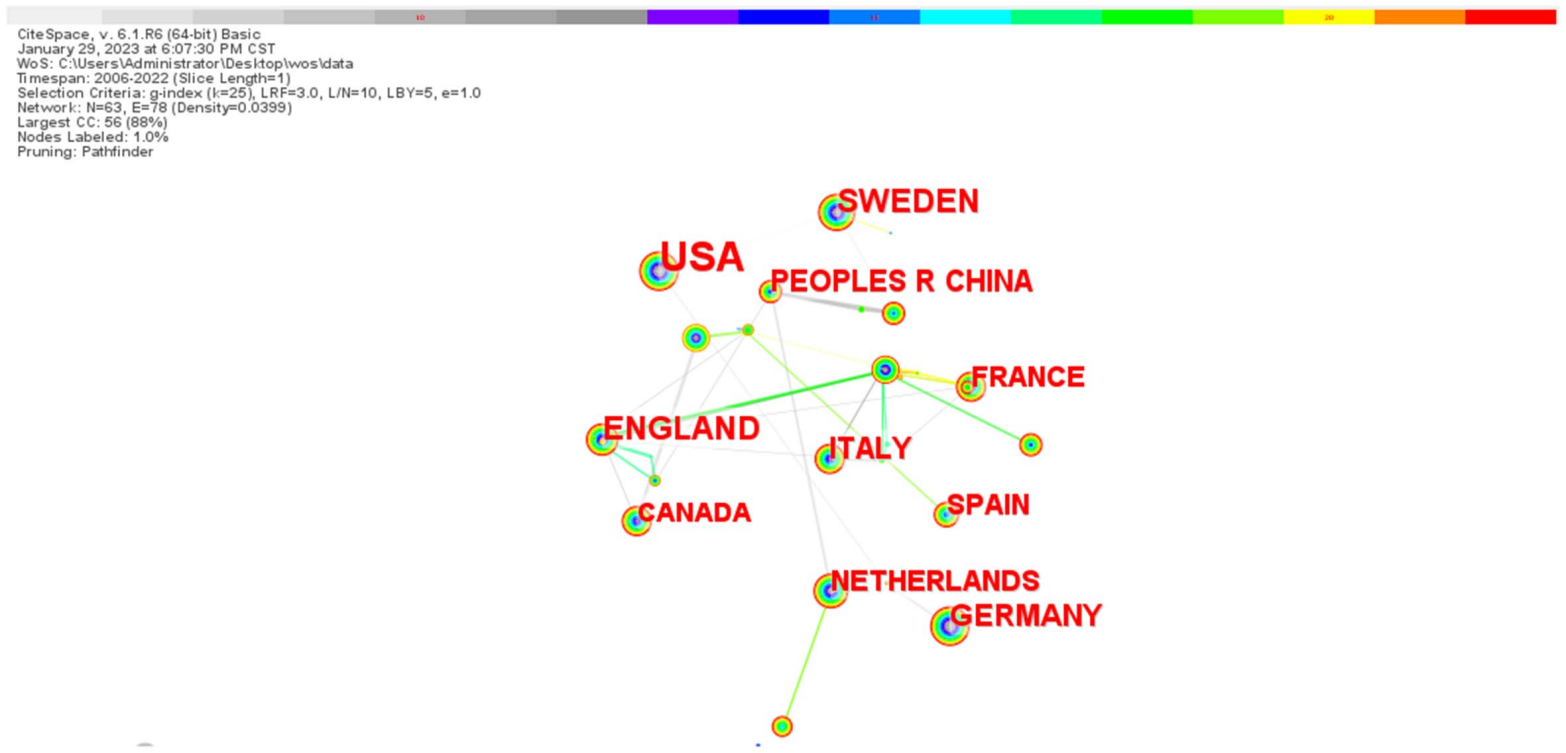
Map of national collaborations on biomarkers and Alzheimer’s disease related research in web of science database (TOP10). The figure illustrates that the size of the nodes corresponds to the number of articles published by each country, while the lines connecting the nodes represent cooperative relationships between countries. Thicker lines indicate closer cooperation. This visualization suggests that the collaboration among the top 10 countries is relatively limited, highlighting the need for enhanced international exchanges in the relevant fields.

In this field, the United States ranked first with 607 documents, followed by the United Kingdom (154), Sweden (141), Germany (113), Italy (95), China (84), the Netherlands (77), and France (76) (Figure 3). The mediational centrality of the research centers was analyzed using the Citespace software, with the United States having a mediational centrality of 0.49, Germany in second place with 0.12, and China with only 0.05. The mediational centrality values are 0.49 in the US, 0.12 in Germany, and 0.05 in China (Figure 4). The major research centers in this field are scattered, mainly concentrated in the US, UK, Canada, Germany, Italy, and China, and the cooperation among the research centers is not very close (Figure 5).

### 3.4 Distribution of research organizations for biomarkers in Alzheimer’s disease

**Figure 6:**
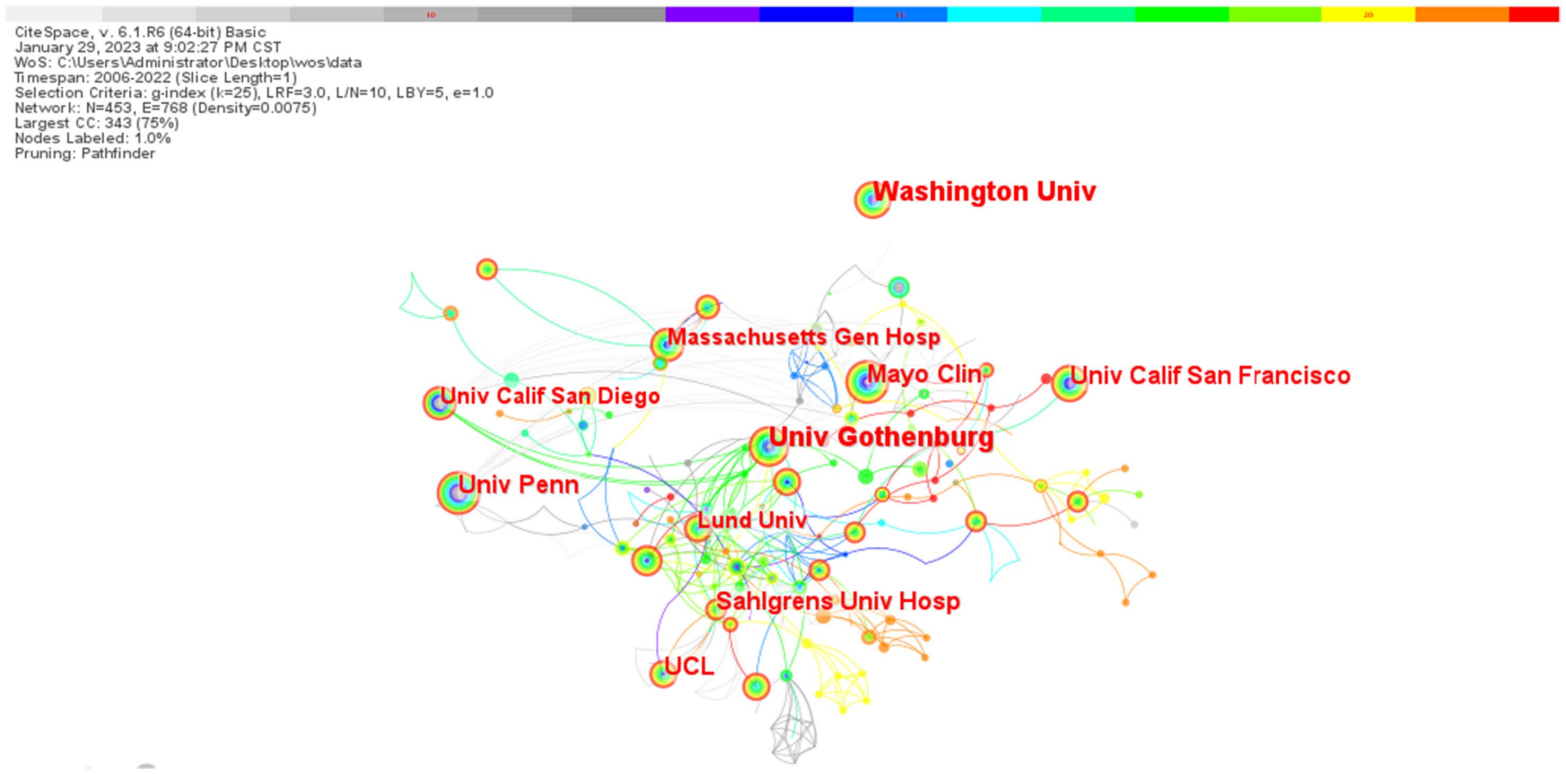
Relationship between biomarkers and Alzheimer’s disease related research organizations in the web of science database (TOP10). The figure illustrates that larger nodes represent organizations with a higher volume of published articles, while thicker lines between nodes indicate stronger collaborative relationships. It suggests that relevant research centers are distributed globally, exhibiting a certain level of mutual cooperation; however, this cooperation is not particularly close.

In terms of the number of publications from research institutions, the University of Washington was at the top of the list (93), followed by the University of Gothenburg and the University of London, with 84 and 63 publications, respectively, while the Mayo Clinic was in the fourth place, with 62 publications (Table 1). By the visualization mapping, there is a certain degree of collaboration between the major research institutions but the closeness of the collaboration needs to be strengthened (Figure 6).

**Table 1:** Institutions publishing biomarker and Alzheimer’s disease related studies in web of science database (TOP10).

Serial number	Institutions	Number of communications
1	Washington Univ	93
2	Univ Gothenburg	84
3	UCL	63
4	Mayo Clin	62
5	Univ Penn	59
6	Univ Calif San Francisco	55
7	Sahlgrens Univ Hosp	48
8	Univ Calif San Diego	40
9	Massachusetts Gen Hosp	38
10	Lund Univ	38

### 3.5 Distribution of authors of studies on biomarkers in Alzheimer’s disease

**Figure 7:**
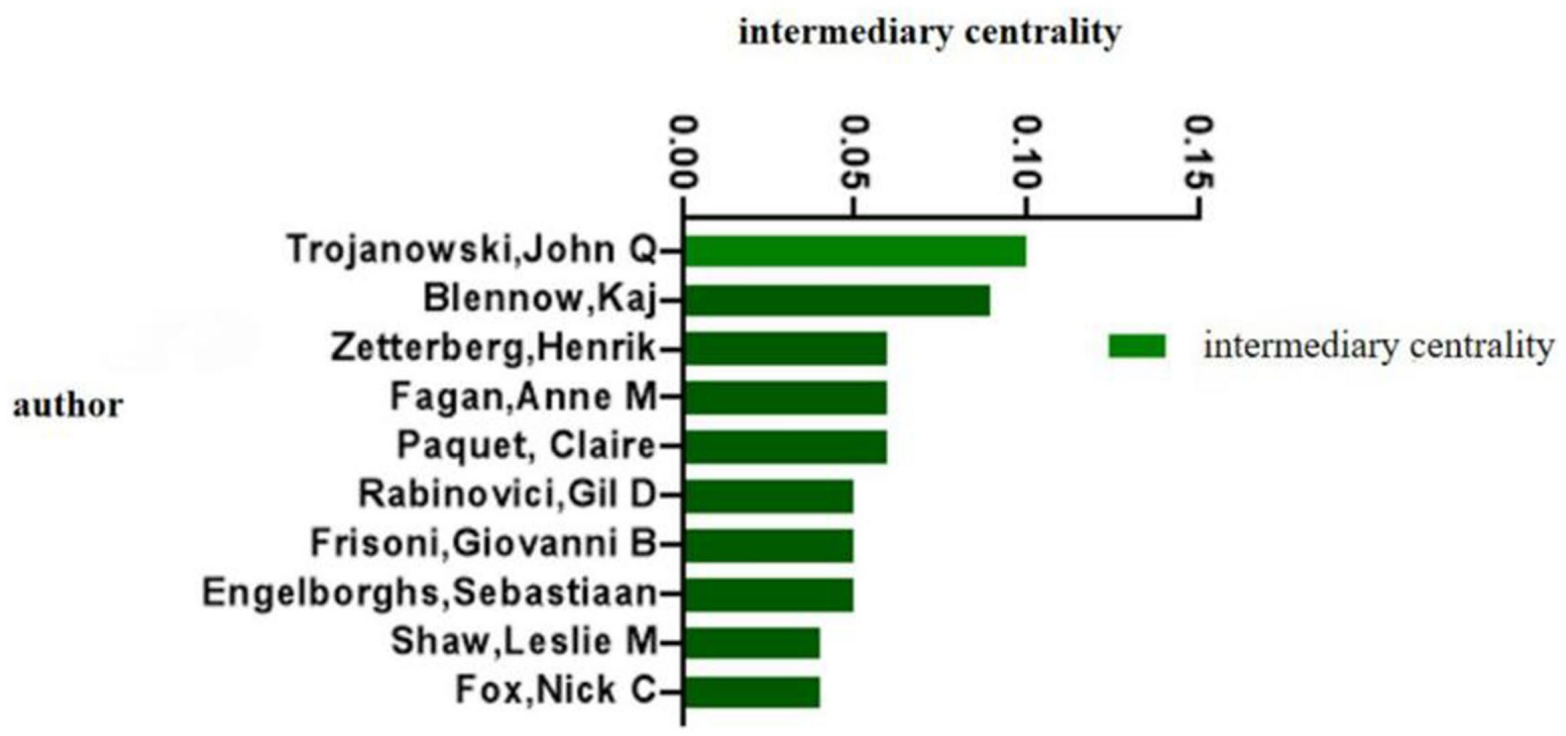
Author mediated centrality of biomarker and Alzheimer’s disease related studies in web of science database (TOP10). Intermediary centrality is a reference used by the Citespace software to measure the importance of a metric, with centrality over 0.01 generally defined as a key metric. Higher values of mediational centrality suggest that the corresponding author is more influential.

**Figure 8:**
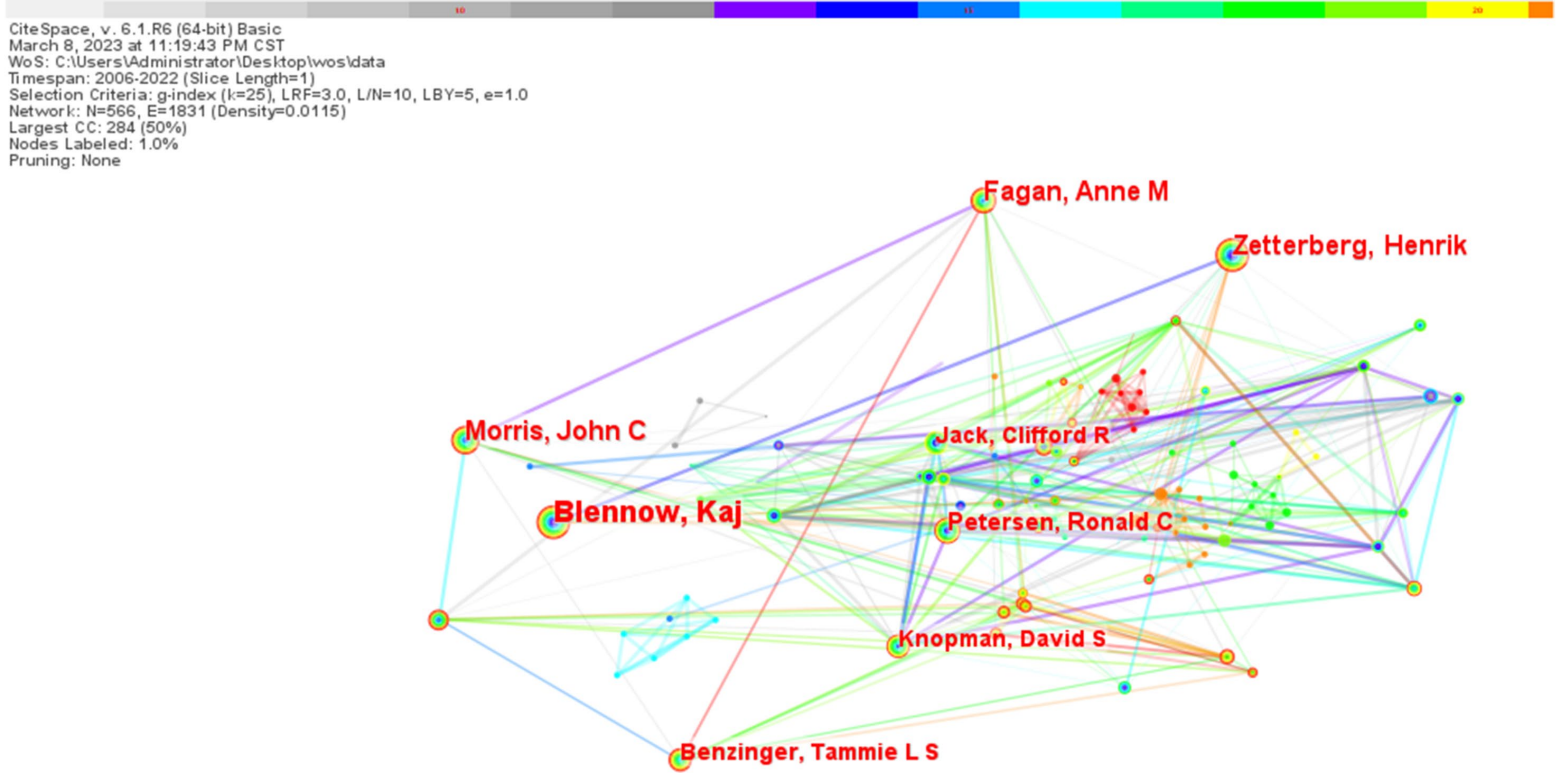
Graph of biomarker and Alzheimer’s disease related study author collaborations in web of science database (TOP10). In the figure, larger nodes represent authors with a greater number of published papers, while thicker lines connecting the nodes indicate a closer collaborative relationship between the two authors.

In terms of the number of publications by research authors, the Swedish scholar Blennow Kaj ranked first with 82 publications, and the second and third ranked scholars were Zetterberg Henrik (78) and Morris John C (64), respectively (Table 2). In terms of mediational centrality, the American scholar Trojanowski John Q ranked the world with 0.1 first place, and the second and third ranked scholars are Blennow Kaj (0.09) and Zetterberg Henrik (0.06), respectively (Figure 7). From the analysis of the visual mapping, the related studies formed two major research centers, one with Blennow Kaj, Fagan, Anne, and Morris John C, and the other with Blennow Kaj and Zetterberg Henrik as the research center, and all the major scholars have a close cooperation relationship with each other (Figure 8).

**Table 2:** Analysis of authors of studies related to biomarkers and Alzheimer’s disease in web of science database (TOP10).

Serial number	Frequency	Years	Author
1	82	2006	Blennow Kaj
2	78	2009	Zetterberg Henrik
3	64	2011	Morris John C
4	54	2007	Fagan Anne
5	37	2011	Petersen Ronald C
6	36	2011	Jack Clifford R
7	32	2011	Benzinger Tammie L S
8	30	2012	Knopman, David S
9	28	2011	Holtzman, David M
10	21	2010	Trojanowski John Q
11	21	2011	Xiong Chengjie

### 3.6 Distribution of co-citations of biomarkers in Alzheimer’s disease

#### 3.6.1 Author co-citation analysis

**Figure 9:**
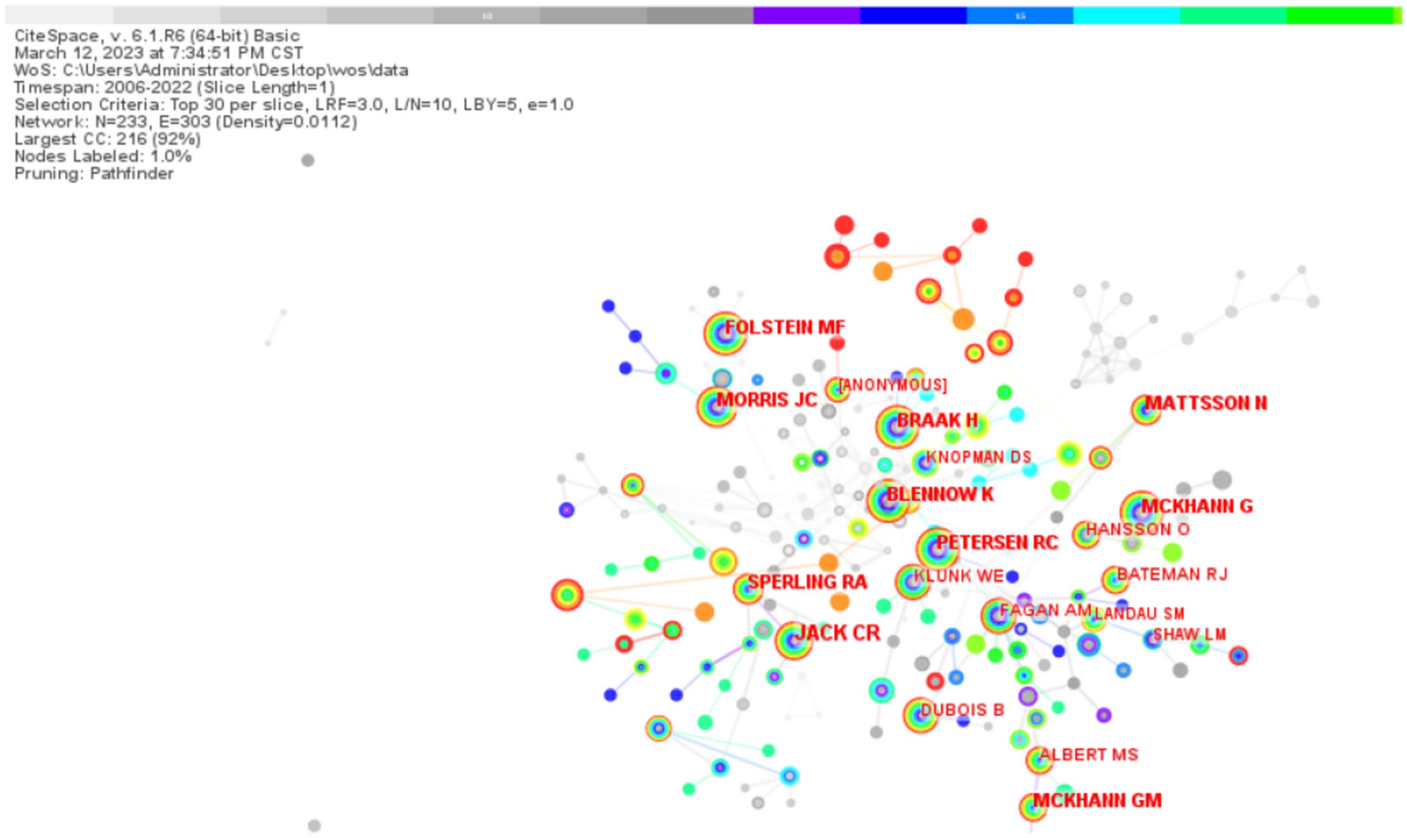
Graph of co-citation relationship between authors of biomarker and Alzheimer’s disease related studies in web of science database (TOP 20). In the figure, the size of each node is directly proportional to the frequency with which the corresponding author is cited.

In the analysis of co-cited authors, the scholar JACK CR ranked the first with 377 citation frequency, and the second to the fifth were MORRIS JC, BLENNOW K, MCKHANN G, and BRAAK H, respectively (Table 3). By the visual mapping analysis, some scholars have a close cooperation relationship with each other, such as JACK CR and MORRIS JC, and some scholars lack a closer connection, such as MCKHANN GM with FOLSTEIN MF (Figure 9).

**Table 3:** Authors of biomarker and Alzheimer’s disease related studies in web of science database co-cited (TOP20).

Serial number	Frequency	Years	Co-cited authors
1	377	2007	JACK CR
2	211	2006	MORRIS JC
3	210	2006	BLENNOW K
4	209	2006	MCKHANN G
5	197	2006	BRAAK H
6	193	2006	PETERSEN RC
7	178	2010	MATTSSON N
8	177	2011	SPERLING RA
9	175	2006	FOLSTEIN MF
10	172	2012	MCKHANN GM
11	157	2009	DUBOIS B
12	124	2007	FAGAN AM
13	116	2012	ALBERT MS
14	113	2010	BATEMAN RJ
15	103	2007	HANSSON O
16	91	2006	KLUNK WE
17	88	2012	LANDAU SM
18	85	2009	SHAW LM
19	82	2009	ANONYMOUS
20	77	2008	KNOPMAN DS

#### 3.6.2 Journal co-citation analysis

**Figure 10:**
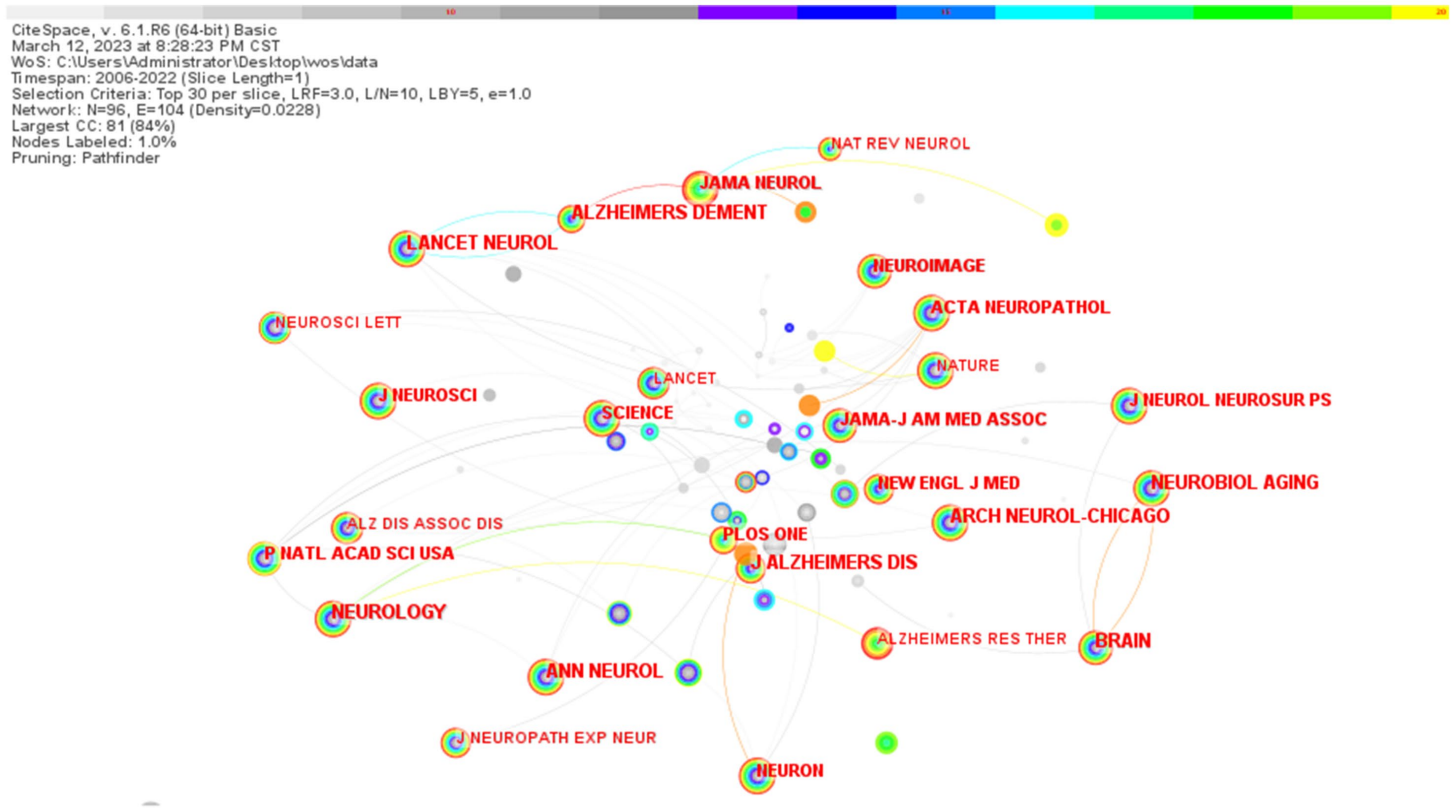
Graph of co-citation relationship between biomarkers and Alzheimer’s disease related research journals in web of science database (TOP20). The larger the node in the figure, the more frequently the journal is cited, and the link represents a collaborative relationship between the two journal.

In terms of journals’ co-citation, NEUROLOGY ranked first with 943 citations, followed by NEUROBIOL AGING, ALZHEIMERS DEMENT, ANN NEUROL, and J ALZHEIMERS DIS (Table 4). Some journals have close citation relationships with each other, such as NEUROIMAGE vs. BRAIN, and some journals lack a co-operative citation relationship with each other, e.g., BRAIN and SCIENCE (Figure 10).

**Table 4:** Co-citation statistics of biomarkers and Alzheimer’s disease related research journals in web of science database (TOP20).

Co-cited journals	Frequency	Years
NEUROLOGY	943	2006
NEUROBIOL AGING	686	2006
ALZHEIMERS DEMENT	674	2007
ANN NEUROL	665	2006
J ALZHEIMERS DIS	645	2007
ARCH NEUROL-CHICAGO	613	2006
LANCET NEUROL	610	2006
BRAIN	563	2007
P NATL ACAD SCI USA	460	2006
PLOS ONE	443	2009
ACTA NEUROPATHOL	403	2006
J NEUROSCI	378	2006
NEUROIMAGE	361	2006
NEURON	350	2006
JAMA NEUROL	343	2014
JAMA-J AM MED ASSOC	339	2006
J NEUROL NEUROSUR PS	334	2006
NEW ENGL J MED	313	2007
SCIENCE	306	2006
NEUROSCI LETT	306	2006

#### 3.6.3 Reference co-citation analysis

In terms of reference co-citation, the top 10 references in terms of citation frequency (Table 5), in which the reference with serial number 1 was published by Jack CR et al. in 2018, and its burst value reached 39.9. Jack CR and his research team accounted for a total of four of the top 10 references in terms of citation frequency.

**Table 5:** Co-citation of biomarker and Alzheimer’s disease related research references in web of science database (TOP10).

Serial number	Author	Co-cited reference titles	Frequency	Years
1	Clifford et al. (2018)	NIA-AA Research Framework: Toward a biological definition of Alzheimer’s disease	117	2018
2	Sperling et al. (2011)	Toward defining the preclinical stages of Alzheimer’s disease: Recommendations from the National Institute	70	2011
3	Shaw et al. (2009)	Cerebrospinal fluid biomarker signature in Alzheimer’s disease neuroimaging initiative subjects	53	2009
4	Jack et al. (2010)	Hypothetical model of dynamic biomarkers of the Alzheimer’s pathological cascade	52	2010
5	Guy et al. (2011)	The diagnosis of dementia due to Alzheimer’s disease: Recommendations from the National Institute on Aging-Alzheimer’s Association workgroups on diagnostic guidelines for Alzheimer’s disease	51	2011
6	Jack et al. (2013)	Tracking pathophysiological processes in Alzheimer’s disease: an updated hypothetical model of dynamic biomarkers	49	2013
7	Dubois et al. (2007)	Research criteria for the diagnosis of Alzheimer’s disease: revising the NINCDS–ADRDA criteria	44	2014
8	Mattsson et al. (2019)	Association of Plasma Neurofilament Light With Neurodegeneration in Patients With Alzheimer Disease	43	2017
9	Olsson et al. (2016)	CSF and blood biomarkers for the diagnosis of Alzheimer’s disease: a systematic review and meta-analysis	36	2016
10	Jack et al. (2016)	A/T/N: An unbiased descriptive classification scheme for Alzheimer disease biomarkers	36	2016

### 3.7 Analysis of research hotspots

#### 3.7.1 Reference co-citation timeline graphs

**Figure 11:**
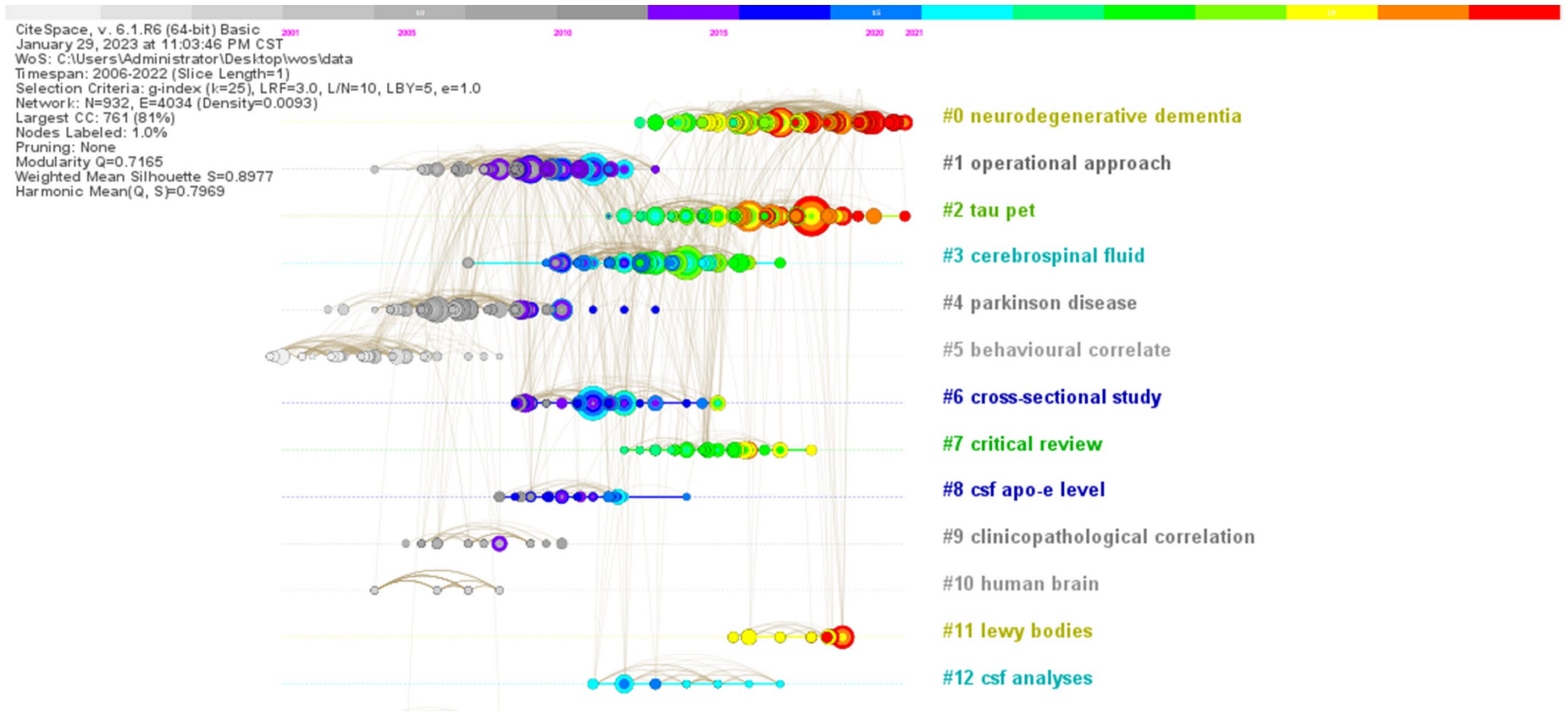
Timeline of co-citation of biomarkers with references from Alzheimer’s disease related studies in the web of science database. The color scale at the top of the figure illustrates different years, ranging from 2006 to 2022, from left to right. The nodes within the graph represent references, with larger nodes indicating a higher frequency of citations. The gradient color transition from the center to the periphery of each node corresponds to the color scale, reflecting the total citation frequency of the literature for each respective year. The horizontal axis position of each node aligns with the time axis, indicating the publication date of the literature from 2006 to 2022. The lines connecting pairs of nodes represent the co-citation relationships between the respective papers; the thickness of these lines correlates with the frequency of co-citation, where thicker lines denote a higher co-citation frequency. Additionally, the color of the lines corresponds to the aforementioned color scale, indicating the initial co-citation year of the two documents.

The results are shown below (Figure 11), where the clusters “neurodegenerative dementia,” “operative approach” and “tau pet” have the highest number of publications, as shown in the timeline graph of reference co-citation using Citespace software. “neurodegenerative dementia,” “operative approach” and “tau pet” had the highest number of publications.

#### 3.7.2 Keyword co-occurrence analysis

**Figure 12:**
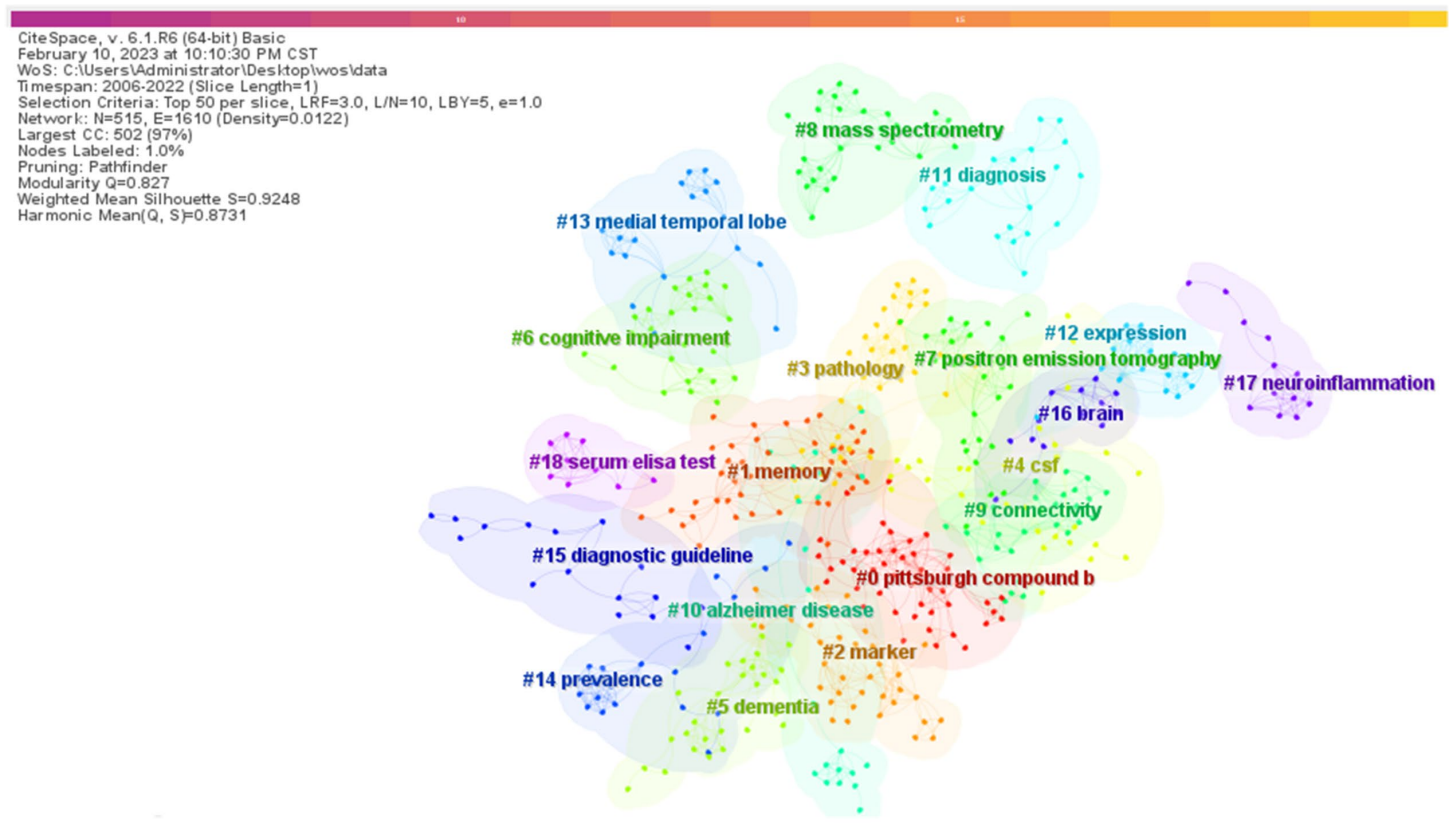
Clustering of keywords for biomarker and Alzheimer’s disease related research in web of science database. # represents different clustering labels.

All keywords were clustered and visualized using Citespace software (using the LLR algorithm), resulting in a total of 18 clusters (Figure 12). The size of the cluster number indicates how many keywords the cluster contains, with the smaller the cluster number indicating the greater the number of keywords it contains. The color corresponding to the cluster area indicates the time of the first co-citation occurrence.

## 4 Discussion

### 4.1 Bibliometric analysis of studies relating biomarkers to Alzheimer’s disease

Between 2006 and 2019, the annual number of publications in the aforementioned field exhibited an upward trend. However, post-2019, while the volume of publications remains significant, there has been a noticeable decline in the overall trend year by year. This suggests that the momentum of research in this field may be weakening since 2019, indicating a slight insufficiency in the research backbone. Nevertheless, this does not imply that the entire research field is falling behind; on the contrary, it may indicate advancements in the quality of research or specific research directions. Therefore, we should maintain an optimistic outlook regarding the changes in research trends, as more high-quality research may be forthcoming, necessitating increased attention to developments within the field. In terms of the number of national publications, the United States leads with 607 articles, which is far ahead of other countries, followed by the United Kingdom (154 articles) and Sweden (141 articles), while China ranks 6th with only 84 articles. In terms of mediocentricity, the mediocentricity of the United States is 0.49, and that of Germany is 0.12, indicating that the United States has a great influence in this research field and is in the absolute leading position, while China’s mediocentricity is only 0.05, which is a big gap between the United States and the first ranked country, and this is the direction that Chinese scholars should strive for in the future. From the perspective of issuing institutions, Washington Univ, Univ Gothenburg, UVL, Mayo Clin, Univ Penn ranked 1st to 5th respectively, among which Washington Univ has a high level in the field of biomarker and Alzheimer’s disease related research, and many of its articles are of high quality, but it lacks a closer cooperation relationship with other institutions. However, there is a lack of close cooperation between this institution and other institutions. However, there is a lack of close collaboration between this institution and other institutions. It is clear that there is a need to strengthen the collaboration between major institutions in the future in order to promote the development of this field, especially for Chinese institutions and scholars. Chinese institutions and scholars should proactively seek collaborations with other countries, such as the United States, and engage with prominent researchers to enhance the influence of biomarker and Alzheimer’s disease-related research. This could involve participating in research projects as sub-centers or conducting original research under the mentorship of leading experts. By actively accumulating experience, Chinese scholars can work toward narrowing the academic gap with leading countries and scholars, thereby strengthening their position in international discourse.

### 4.2 Analysis of research hotspots related to biomarkers and Alzheimer’s disease

Keyword clustering analysis can reflect the research hotspots in the field to a certain extent.

#### 4.2.1 Cluster 0: Pittsburgh compound B

Early diagnosis of Alzheimer’s disease is a major challenge, which has led to the development of imaging reagents in the field of Alzheimer’s disease, one of which is Pittsburgh Compound B (PiB). Animal studies have demonstrated (Bacskai et al., 2003) that PiB rapidly crosses the brain barrier to mark amyloid deposits, which can be used in the diagnosis of patients with Alzheimer’s disease, and that the degree of PiB binding may correlate linearly with the amount of neuritic plaque formation (Dana et al., 2012). It has also been shown that different parts of the brain may have different abilities to bind PiB (Tina et al., 2012). In cognitively normal older adults, PiB PET brain scans can still be positive, suggesting the presence of preclinical Alzheimer’s disease (Niedowicz et al., 2012). For patients with Alzheimer’s disease, white matter PiB uptake was significantly higher than cortical, and the frontal, occipital and posterior cingulate gyrus were more capable of binding PiB (Beckett et al., 2012). There is a plateau in PiB deposition in patients with Alzheimer’s disease. Alzheimer’s disease progression may be positively associated with it, but after entering the plateau phase its deposition is no longer exacerbated by disease progression. In patients with disseminated Alzheimer’s disease and mild cognitive impairment, different subgroups of amyloid *β* peptide (Aβ) possess different PiB binding capacities. The need to explore the effects of PiB imaging and to find better imaging reagents has long been a major driver of research in the field of Alzheimer’s disease.

#### 4.2.2 Cluster 2: markers

In terms of time course, there is a wealth of research on biomarkers in Alzheimer’s disease, particularly fuelled by the discovery of biomarkers in cerebrospinal fluid. Discoveries such as the discovery of Tau protein and Aβ in cerebrospinal fluid, the first use of ELISA to measure T-Tau and phosphorylated Tau, and the combination of Aβ42 and Aβ40 in cerebrospinal fluid to improve the diagnostic accuracy of AD have been gradually revealed (Blennow and Zetterberg, 2018). Currently, many scholars are working to find better biomarkers or more accurate prediction methods for existing markers in patients with AD or pre-AD (Ossenkoppele et al., 2022). Compared to cerebrospinal fluid biomarkers, blood-based biomarkers have lagged behind (Teunissen et al., 2021), and some blood-based biomarkers such as neurofilament light chains and glial fibrillary acidic protein may have the potential to provide information on the progression of neurodegenerative diseases and to monitor the effects of treatment. Cerebrospinal fluid biomarkers such as NSE, VLP-1, HFABP and YKL-40 may be associated with Alzheimer’s disease. In conclusion existing biomarkers in AD diagnosis has achieved some research results, but expensive and relatively invasive operation, how to have fast and cost-effective biomarkers to diagnose, promote artificial intelligence algorithms (Chun-Hung Chang, 2021) and how to define the role of biomarkers and other biomarkers in the diagnosis of AD at the individual level is a major driving direction for future research.

### 4.3 Limitations of the article

This paper exclusively utilized the Web of Science database and focused solely on literature published in English. Consequently, high-quality literature published in Chinese or other languages may have been overlooked, which introduces a potential bias. Nevertheless, the literature included in the Web of Science database is generally recognized for its high quality and offers a relatively comprehensive overview of research areas pertaining to biomarkers and Alzheimer’s disease. Therefore, while the findings presented in this paper are subject to some bias, they remain relatively reliable overall.

### 4.4 Summary and outlook

This article summarizes the literature on biomarkers and Alzheimer’s disease over a 17-year period from 2006 to 2022, and analyses the global research trends and hotspots in the field. Alzheimer’s disease is a complex degenerative disease of the central nervous system that is difficult to identify at an early stage of clinical development and has poor therapeutic effects. The progress of research is slow, and the process of translating laboratory results into clinical practice is slow and challenging. However, because of these challenges, further in-depth investigation and resolution of these challenges is the future trend in this field.

## 5 Conclusion

Alzheimer’s disease is a complex and clinically challenging condition that is often difficult to recognize in its early stages and is poorly treated as it progresses. Currently, biomarkers such as Aβ1-40, Aβ1-42, T-tau, and P-tau serve as significant foundations for the early diagnosis of Alzheimer’s disease by clinicians. Although there has been a quantitative decline in research related to biomarkers for Alzheimer’s disease in recent years, this does not necessarily indicate a substantial decrease in research quality. On the contrary, it is possible that more promising biomarkers for the prediction and diagnosis of Alzheimer’s disease may emerge in the future. The identification of potential biomarkers that are more clinically relevant is a common objective among major studies. Strengthening collaboration among countries, leading research institutions, and scholars—through the development of mutually beneficial relationships, the establishment of research sub-centers, and opportunities for leading scholars to pursue further training and study— is critical for facilitating the early discovery of more promising biomarkers.
